# The effects of martial arts participation on mental and psychosocial health outcomes: a randomised controlled trial of a secondary school-based mental health promotion program

**DOI:** 10.1186/s40359-019-0329-5

**Published:** 2019-09-11

**Authors:** Brian Moore, Dean Dudley, Stuart Woodcock

**Affiliations:** 1 Charles Sturt University, School of Teacher Education Faculty of Arts and Education, Panorama Avenue, Bathurst, NSW 2795 Australia; 2 Macquarie University, Department of Educational Studies Faculty of Human Sciences, Balaclava Road, Macquarie, NSW 2109 Australia; 3 Griffith University, School of Education and Professional Studies Faculty of Arts, Education, and Law, Brisbane, QLD 4122 Australia

**Keywords:** Mental health, Martial arts, Resilience, Self-efficacy, Preventative medicine, Alternative and complimentary therapies

## Abstract

**Background:**

Mental health problems are a significant social issue that have multiple consequences, including broad social and economic impacts. However, many individuals do not seek assistance for mental health problems. Limited research suggests martial arts training may be an efficacious sports-based mental health intervention that potentially provides an inexpensive alternative to psychological therapy. Unfortunately, the small number of relevant studies and other methodological problems lead to uncertainty regarding the validity and reliability of existing research. This study aims to examine the efficacy of a martial arts based therapeutic intervention to improve mental health outcomes.

**Methods/design:**

The study is a 10-week secondary school-based intervention and will be evaluated using a randomised controlled trial. Data will be collected at baseline, post-intervention, and 12-week follow-up. Power calculations indicate a maximum sample size of *n* = 293 is required. The target age range of participants is 11–14 years, who will be recruited from government and catholic secondary schools in New South Wales, Australia. The intervention will be delivered in a face-to-face group format onsite at participating schools and consists of 10 × 50–60 min sessions, once per week for 10 weeks. Quantitative outcomes will be measured using standardised psychometric instruments.

**Discussion:**

The current study utilises a robust design and rigorous evaluation process to explore the intervention’s potential efficacy. As previous research examining the training effects of martial arts participation on mental health outcomes has not exhibited comparable scale or rigour, the findings of the study will provide valuable evidence regarding the efficacy of martial arts training to improve mental health outcomes.

**Trial registration:**

Australian New Zealand Clinical Trials Register ACTRN12618001405202. Registered 21st August 2018.

## Background

Mental health problems are a significant social issue that have multiple consequences; ranging from personal distress, disability, and reduced labour force participation; to wider social and economic impacts. The annual global cost of mental health problems was estimated as $USD 2.5 trillion by the World Health Organisation [1]; and the annual cost of mental illness in Australia has been estimated as $AUD 60 billion [2]. These costs are projected to increase 240% by 2030 [1].

However, for a variety of reasons including stigmatisation of mental health and the cost and poor availability of mental health treatment, many individuals do not seek assistance for mental health problems [3]. Consequently, it is important to consider the application of alternative and complimentary therapies regarding mental health treatment. Martial arts training may be a suitable alternative, as it incorporates unique characteristics including an emphasis on respect, self-regulation and health promotion. Due to this, martial arts training could be viewed as a sports-based mental health intervention that potentially provides an inexpensive alternative to psychological therapy [4]. However, the efficacy of this approach has received little research attention [5].

Existing martial arts research has mostly focused on the physical aspects of martial arts, including physical health benefits and injuries resulting from martial arts practice [6], while few studies have examined whether martial arts training addressed mental health problems or promoted mental health and wellbeing. Several studies report that martial arts training had a positive effect reducing symptoms associated with anxiety and depression. For example: (a) training in tai-chi reduced anxiety and depression compared to a non-treatment condition [7], (b) karate students were less prone to depression compared to reported norms for male college students [8], and (c) a study examining a six-month taekwondo program reported significantly reduced anxiety [9]. Similarly, several studies report martial arts training promotes characteristics associated with wellbeing including: (a) a group of female participants reported higher self-concept compared to a comparison group after studying taekwondo for 8 weeks [10], and (b) a six-month taekwondo program found increased self-esteem following the intervention [9].

A recent meta-analysis examining the effects of martial arts training on mental health examined 14 studies and found that martial arts training had a positive effect on mental health outcomes (Moore, B., Dudley, D. & Woodcock, S. The effect of martial arts training on mental health outcomes: a systematic review and metaanalysis, Under review). The study found that martial arts training had a medium effect size regarding reducing internalising mental health problems, such as anxiety and depression; and a small effect size regarding increasing wellbeing.

However, despite generally positive findings the research base examining the psychological effects of the martial arts training exhibits significant methodological problems [11, 12]. These include definitional and conceptual issues, a reliance on cross-sectional research designs, small sample sizes, self-selection effects, the use of self-report measures without third party corroboration, absence of follow-up measures, not accounting for demographic differences such as gender, and issues controlling for the role of the instructor. These issues may limit the generalisability of findings and suggest uncertainty regarding the validity and reliability of previous research.

This study seeks to examine the relationship between martial arts training and mental health outcomes, while addressing the methodological limitations of previous studies. The intervention examined by the study is a bespoke programme based primarily on the martial art taekwondo and incorporating psycho-education developed for the intervention. Importantly, this study aims to examine the efficacy of a martial arts based therapeutic intervention to improve mental health outcomes.

## Methods/design

### Study design

This study is a 10-week secondary school-based intervention and will be evaluated using a randomised controlled trial. Ethics approval has been sought and obtained from an Australian University Human Research Ethics Committee, the New South Wales (NSW) Department of Education, and the Catholic Education Diocese of Parramatta. The study is registered with the Australian and New Zealand Clinical Trials Registry (ACTRN12618001405202). The study protocol was also reviewed externally by school psychologists employed by the NSW Department of Education.

Researchers will conduct baseline assessments at participating schools after the initial recruitment processes. Following baseline assessments and randomisation, the intervention group will receive the intervention after which post-intervention assessment will be conducted. A 12-week post-intervention (follow-up) assessment will also be conducted. The control group will receive the same intervention program after the first post-intervention assessment and will not be measured at follow-up. The design, conduct and reporting of this study will adhere to the Consolidation Standards of Reporting Trials (CONSORT) guidelines for a randomised controlled trial [13]. Participants and caregivers will provide written informed consent.

### Sample size calculation

Power calculations were conducted to determine the sample size required to detect changes in mental health related outcomes resulting from martial arts training. Statistical power calculations assumed baseline-post-test expected effect size gains of *d* = 0.3, and were based on 90% power with alpha levels set at *p* < 0.05. The minimum completion sample size was calculated as *n* = 234 (intervention group: *n* = 117, control group: *n* = 117). As participant drop-out rates of 20% are common in randomised controlled trials [14], the maximum proposed sample size was *n* = 293 (intervention group: *n* = 147, control group: *n* = 146).

### Recruitment and study participants

To be eligible to participate in the study, schools must be government or catholic secondary schools in NSW, Australia. All eligible schools (*n* = 140) will be sent an initial email with an invitation to participate in the study. Schools that respond to the initial email will be pooled and receive a follow up call in random order from the project researchers to discuss whether they would like to participate in the study. The first five schools that demonstrate interest will then be recruited into the study.

Inclusion criteria for participation in the study includes: (a) participants are currently enrolled in grades 7 or 8, and (b) participants are within an age range 11–14 years. Exclusion criteria: concurrent martial arts training will exclude participation in the study, however prior experience of martial arts training is not an exclusion criteria. All students at participating schools who meet these criteria will be invited to participate in the study. Participant and caregiver information and consent forms will be provided to students. Two follow-up letters will be sent subsequently at 2 week intervals. Students who respond to the invitation will be pooled and randomly allocated into the study, or not included in the study.

**Fig. 1:**
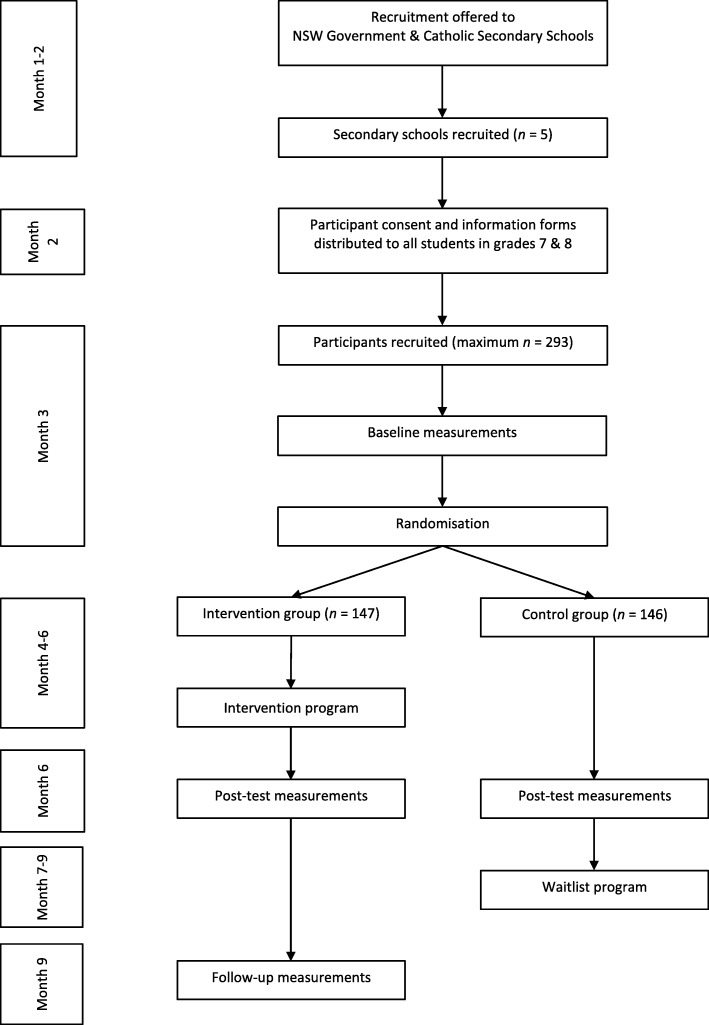
Flowchart of study

Randomisation into intervention and control group will occur after baseline assessments. A simple computer algorithm will be used to randomly allocate participants into intervention or control groups. This will be performed by a researcher not directly involved in the study. Figure 1 provides a flowchart of the timeline for the study.

### Intervention design

#### Intervention description

The intervention will be delivered in a face to face group format onsite at participating schools. The intervention will be 10 × 50–60 min sessions, once per week for 10 weeks. Each intervention session will include:

- Psycho-education – guided group based discussion. Topics include respect, goal-setting, self-concept and self-esteem, courage, resilience, bullying and peer pressure, self-care and caring for others, values, and, optimism and hope;
- Warm up – including jogging, star jumps, push ups, and sit ups;
- Stretching – including hamstring stretch, triceps stretch, figure four stretch, butterfly stretch, lunging hip flexor stretch, knee to chest stretch, and standing quad stretch; and,
- Technical practice – including stances, blocks, punching, and kicking.

Additionally, intervention sessions intermittently include (alternated throughout the program):

(e) Patterns practice – a pattern is a choreographed sequence of movements consisting of combinations of blocks, kicks, and punches performed as though defending against one or more imaginary opponents;
(f) Sparring – based on tai-chi sticking hands exercise (which has been included as an alternative to traditional martial arts sparring); and
(g) Meditation – based on breath focusing exercise.

In the final session the intervention will conclude with a formal martial arts grading where participants will be awarded a yellow belt subject to demonstration of martial arts techniques (stances, blocks, punching, and kicking) and the pattern learnt during the program. While it is desirable for participants to attend all 10 sessions to achieve intervention dose, it is unrealistic to assume all sessions will be attended. Research has suggested that determining an adequate intervention dose in health promotion programmes can be based on level of participation and whether participants did well [15]. In the current study intervention dose will be assumed if participants successfully complete the formal grading and are awarded a yellow belt. It is important to note that aggressive physical contact is not part of the intervention program. The intervention will be delivered by a (1) registered psychologist with minimum 6 years’ experience, and (2) 2nd Dan/level black-belt taekwondo instructor with minimum 5 years’ experience. Materials used during the intervention will include martial arts belts (white and yellow), and martial arts training equipment (for example strike paddles, strike shields).

#### Theoretical framework

The intervention development and implementation will be based on a traditional martial arts model, dichotomous health model, and social cognitive theory. Research examining the relationship between the martial arts and mental health has typically used a bipartite model [16] which distinguishes between traditional and modern martial arts. The intervention is based on a traditional martial arts perspective, which emphasizes the non-aggressive aspects of martial arts including psychological and philosophical development [17].

The absence of an explicit health model is a significant methodological limitation of previous research examining the mental health outcomes of martial arts training. The dominant models of mental health are based on the homeostatic assumption that normal health reflects the tendency towards a relatively stable equilibrium; and that the dysregulation of homeostatic processes causes ill-health [18]. These models can be defined dichotomously as having a: (1) pathological basis (deficit model) which refers to the presence or absence of disease based symptoms such as depression or anxiety; and (2) wellbeing basis (strengths model) which refers to the presence or absence of beneficial mental health characteristics such as resilience or self-efficacy. While considering both aspects of the mental health continuum, this study was particularly interested in the strengths model and examined the wellbeing characteristics of resilience and self-efficacy.

Social cognitive theory suggests that knowledge can be acquired through the observation of others in the context of social interactions, experiences, and media influences; and explains human behaviour in terms of continuous reciprocal interaction between personal cognitive, behavioural, and environmental influences [19]. The theory is useful for explaining the learning processes in the martial arts, which include: (a) modelling – where learning occurs through the observation of models; (b) outcome expectancies – to learn a modelled behaviour the potential outcome of that behaviour must be understood (for example, the anticipation of rewards or punishment); and (c) self-efficacy – the extent to which an individual believes that they can perform a behaviour required to produce a particular outcome [19].

The study’s theoretical framework incorporating a traditional martial arts model, dichotomous health model and social cognitive theory facilitates examination of the effects of martial arts training on mental health, ranging from mental health problems to factors associated with wellbeing such as resilience and self-efficacy. Further, the framework may determine the efficacy of martial arts training as an alternative mental health intervention that improves mental health outcomes.

### Outcomes

Evaluation of the intervention program will involve a variety of standardised psychometric instruments to report on mental health related outcomes. Instruments include the Strengths and Difficulties Questionnaire (SDQ) [20], Child and Youth Resilience Measure (CYRM) [21], and Self-Efficacy Questionnaire for Children (SEQ-C) [22]. All outcome time-points will be examined 1 week pre-intervention, 1 week post-intervention, and 12-week post-intervention (follow-up).

#### Behavioural and emotional difficulties

The primary outcome measured by the SDQ will be mean total difficulties. Additionally, the SDQ will measure the following secondary outcomes: emotional difficulties, conduct difficulties, hyperactivity difficulties, peer difficulties, and pro-social behaviour.

Total difficulties was selected as a primary outcome as it provides an overview of participants’ psychological problems. The SDQ scale is a commonly used psychometric screening tool recommended for use by the Australian Psychological Society [23] and has been normed for the Australian population.

#### Resilience

The primary outcome measured by the CYRM will be mean total resilience. Additionally, the CYRM will measure the following secondary outcomes: individual capacities and resources, relationships with primary caregivers, and contextual factors.

Resilience was selected as a primary outcome as it is a current focus of research regarding psychological strengths, but has not been examined regarding the effect of martial arts training. The CYRM-28 was used in the study as it efficiently operationalises the theoretical aspects of resilience in a valid and reliable manner, but is shorter than comparable scales (for example the Resilience Scale for Children and Adolescents [24]).

#### Self-efficacy

The primary outcome measured by the SEQ-C will be mean total self-efficacy. Additionally, the SEQ-C will measure the following secondary outcomes: academic self-efficacy, social self-efficacy, and emotional self-efficacy.

Self-efficacy was selected as a primary outcome as this operationalised a relevant component of social cognitive theory, which is important regarding the hypothesised learning processes in the intervention. The SEQ-C was used in the study as it operationalises self-efficacy for adolescents in an educationally relevant context.

### Statistical methods

Statistical analysis of the primary and secondary outcomes will be conducted using SPSS statistics version 25 (IBM SPSS Statistics, 2017) and alpha levels will be set at *p* < 0.05.

The collected psychometric test data will be consolidated into subscale variables using factor analysis and the internal consistency of each variable will be examined to determine reliability. Items to be included in the scale variables will be added and computed to create composite scores. Repeated measures univariate analysis of variance (ANOVA), and multivariate analysis of variance (MANOVA) will primarily be used to analyse test data. Ordinal regression will be used to analyse test data based on psychometric measures using a 3-point Likert scale. Interpretation of effect sizes will reflect Cohen’s suggested small, medium, and large effect sizes, where partial eta squared sizes are equal to 0.10, 0.25, and 0.40 respectively [25].

Age, school grade level, sex, socio-economic status and cultural background will be included as covariates in the analysis.

## Discussion

The primary aim of this study is to evaluate the training effects of martial arts participation on mental health outcomes. The study will use a randomised controlled trial of secondary school aged participants.

Previous studies examining the impact of martial arts training on mental health and wellbeing have found positive results, which has also been confirmed by a systematic review and meta-analysis. Results have included martial arts training reducing symptoms associated with anxiety and depression; and promoting characteristics associated with wellbeing. However, the small number of relevant studies and noted methodological problems lead to uncertainty regarding the validity and reliability of existing research.

The current study utilises a robust design with baseline, post-test and follow-up measures to examine the views of participants and includes a rigorous evaluation process using quantitative data to explore the program’s potential efficacy. This is a clear strength of this study and is important due to the study’s multi-site delivery. The current study has not used a qualitative approach which is a limitation of the research. Qualitative work is planned for future research to explore issues such as mechanism of impact.

### Conclusion

The findings of this study will provide valuable evidence regarding the training effects of martial arts participation on mental health outcomes, and information for research groups looking for alternative or complementary psychological interventions. To our knowledge, no previous studies have reported the training effects of martial arts participation on mental health outcomes on a scale comparable to the current study while maintaining a similarly robust design and rigorous evaluation process. This study has the potential to change public health policy, and school-based policy and practice regarding management of mental health outcomes and enhance a range of health promoting behaviours in schools.

## Data Availability

The datasets used and/or analysed during the current study will be available from the corresponding author on reasonable request.

## Abbreviations

$AUD: Australian dollar
$USD: United States dollar
ACTRN: Australian New Zealand Clinical Trials Registry Number
ANOVA: Analysis of variance
CONSORT: Consolidation standards of reporting trials
CYRM: Child and youth resilience measure
MANOVA: Multivariate analysis of variance
NSW: New South Wales
SDQ: Strengths and difficulties questionnaire
SEQ-C: Self-efficacy questionnaire for children
SPSS: Statistical packages for the social sciences
